# Low-level BTZ-043 resistance in *Mycobacterium tuberculosis* and cross-resistance to bedaquiline and clofazimine

**DOI:** 10.5588/ijtldopen.25.0301

**Published:** 2025-10-10

**Authors:** A. Ghodousi, I. Iannucci, F. Saluzzo, J. Dreisbach, S. Mirold-Mei, M. Hoelscher, D.M. Cirillo

**Affiliations:** ^1^ Vita-Salute San Raffaele University, Milan, Italy;; ^2^ IRCCS San Raffaele Scientific Institute, Milan, Italy;; ^3^ Institute of Infectious Diseases and Tropical Medicine, LMU University Hospital, LMU Munich, Munich, Germany;; ^4^ German Center for Infection Research (DZIF), Partner Site Munich, Munich, Germany;; ^5^ Fraunhofer Institute for Translational Medicine and Pharmacology ITMP, Immunology, Infection and Pandemic Research, Munich, Germany;; ^6^ Unit Global Health, Helmholtz Zentrum München, German Research Center for Environmental Health (HMGU), Neuherberg, Germany.

**Keywords:** tuberculosis, multidrug-drug resistant, extensively drug-resistant, MDR-TB, WGS, MIC

## Abstract

**BACKGROUND:**

Multidrug- and extensively drug-resistant strains of *Mycobacterium tuberculosis* *complex* (MTBC) remain a significant global health challenge. This study investigates resistance mechanisms to BTZ-043, a novel decaprenylphosphoryl-β-D-ribose 2′-epimerase (DprE1) inhibitor, and its potential cross-resistance with bedaquiline (BDQ) and clofazimine (CFZ).

**METHODS:**

BTZ-043-resistant mutants were generated in *M. tuberculosis* H37Rv by serial exposure to escalating drug concentrations. Minimum inhibitory concentrations (MICs) for BTZ-043 were determined for 130 wild-type strains, including 60 H37Rv independent cultures and 70 diverse clinical isolates, plus 33 non–wild-type clinical strains with known BDQ susceptibility. MICs were correlated with whole-genome sequencing (WGS) data to identify genetic factors underlying resistance.

**RESULTS:**

The MIC distribution for clinical MTBC strains was similar to the reference strain, with a mode of 0.002 μg/mL. WGS of resistant mutants revealed mutations in *dprE1* and *Rv0678*. *Rv0678* and *dprE1* mutations resulted in 4- to 8-fold and >1,000-fold increase in MIC compared with the reference mode, respectively. Sequential clinical strains from BDQ-treated patients showed increased MICs and *Rv0678* mutations, indicating low-level cross-resistance. However, *Rv0678* mutations in BDQ-susceptible strains did not affect BTZ-043 MICs.

**CONCLUSION:**

*Rv0678* mutations confer low-level cross-resistance to BTZ-043, BDQ, and CFZ, with variable effects on susceptibility. These findings highlight the complexity of resistance mechanisms and the need for ongoing surveillance and early resistance assessments in drug development.

TB remains a significant global health challenge, exacerbated by the emergence of multidrug-resistant (MDR-TB) and extensively drug-resistant (XDR-TB) strains of *Mycobacterium tuberculosis* *complex* (MTBC).^1^ This rise in drug resistance has intensified the need for novel therapeutic approaches targeting essential bacterial processes. One such target is decaprenylphosphoryl-β-D-ribose 2′-epimerase (DprE1), an enzyme crucial for the biosynthesis of arabinogalactan, a major component of the mycobacterial cell wall.^2,3^ Inhibition of DprE1 disrupts cell wall synthesis, leading to bactericidal effects. Benzothiazinones (BTZs), a class of compounds targeting DprE1, exhibit potent activity against both drug-susceptible and drug-resistant MTBC strains,^2–4^ with BTZ-043 being the most advanced candidate currently in phase 2B clinical trials.^5,6^ BTZ-043 is developed through a partnership between LMU Klinikum (Munich, Germany), the Hans-Knöll-Institute (Jena, Germany), and the German Center for Infection Research (Braunschweig, Germany), with phase 2 trials conducted by the PanACEA consortium.^6^ It is the first TB drug candidate to be exclusively developed by academia. BTZ-043 targets DprE1 and is the first candidate from the benzothiazinone class of drugs.^3^ It is highly lipophilic, with 95% of the drug bound to plasma proteins, and exists primarily as the Meisenheimer complex metabolite (M2) in human plasma, which can revert to the parent compound in the presence of oxygen. In vitro drug–drug interaction assessments have been challenging due to this property. Degradation assays indicate that the M1 metabolite is 500 times less active than the parent compound against *M. tuberculosis*.^6^ Understanding the mechanisms of resistance to BTZ-043 is essential for optimising its clinical use and developing effective combination therapies. Prior research has shown that mutations in *Rv0678* confer low-level resistance to DprE1 inhibitors (quabodepistat and A7371).^7–9^

This study aimed to characterise BTZ-043 resistance by generating resistant mutants in vitro and assessing the impact of these mutations on drug susceptibility and cross-resistance to bedaquiline (BDQ) and clofazimine (CFZ).

## METHODS

### Selection of resistance-associated variants

BTZ-043-resistant mutants were generated by exposing the *M. tuberculosis* H37Rv ATCC 27294 parental strain to escalating concentrations of BTZ-043 over multiple passages. Briefly, the strain was cultured in 10 mL of 7H9 media supplemented with 10% OADC (oleic acid, albumin, dextrose, and catalase), starting at a CFU of 1–1.5 × 10^7^/mL (0.5 McFarland), and this procedure was repeated in five independent cultures.^10^ Following serial passage, cultures were plated on Middlebrook 7H10 agar containing BTZ-043, and resistant colonies were selected for further analysis. Minimum inhibitory concentration (MIC) determinations for the *in vitro*–selected BTZ-043–resistant mutants against BDQ and CFZ were performed using the MGIT960 system, as previously described.^11^

### Whole-genome sequencing and data analysis

Whole-genome sequencing (WGS) was performed on the potential mutants to identify genetic alterations associated with resistance, as described previously.^12^ Briefly, genomic DNA (gDNA) was extracted from cultures grown in Middlebrook 7H9 broth supplemented with OADC. Following the lysis protocol, the extracted gDNA was purified using the semi-automated Maxwell 16 Cell DNA Kit (Promega) according to the manufacturer’s instructions. The purified gDNA was subsequently sequenced on an Illumina NextSeq 500 platform with 150-bp read lengths using the Nextera XT DNA Library Preparation Kit (Illumina). Data analysis was performed using the MTBseq pipeline^13^ in low-frequency detection mode to identify acquired variants relative to the parental strain.

### MIC determination to obtain the wild-type BTZ-043 MIC distribution

A panel of 130 wild-type strains including 60 H37Rv independent cultures, and 70 phylogenetically diverse MTBC clinical isolate (L1–L6, *M. bovis* and *M. canettii*) underwent BTZ-043 MIC determination to obtain the wild-type BTZ-043 MIC distribution using broth micro-dilution in Middlebrook 7H9 broth according to the European Committee on Antimicrobial Susceptibility Testing (EUCAST) MIC testing protocol.^10^ Briefly, the MTBC isolates were tested against serial two-fold dilutions of BTZ-043, ranging from 0.000125 to 0.016 μg/mL, in Middlebrook 7H9 broth. Plates were read on the 7th, 10th, and 14th day of incubation using an inverted mirror. MIC was determined as the lowest concentration of the agent where no visible growth is observed (in the presence of visible growth of Growth Control 1%). Since no established quality control range values for BTZ-043 are currently available, isoniazid (INH) MICs were also determined within the EUCAST quality control range as a reference.

### BTZ-043 MIC testing of clinical strains carrying Rv0678 mutations

To evaluate the impact of *Rv0678* mutations on BTZ-043 susceptibility, we analysed sequential clinical strains from patients (n = 6) in Pakistan who had previously undergone BDQ-containing treatment regimens, as reported previously.^14^ These patients exhibited increased MICs for BDQ and CFZ, accompanied by the emergence of *Rv0678* mutations. We assessed whether these strains had developed resistance or reduced susceptibility to BTZ-043 by determining MICs in duplicate and comparing them to baseline levels. Furthermore, a panel of 16 BDQ-resistant clinical strains harbouring various *Rv0678* mutations – including frameshifts, premature stop codons, and amino acid substitutions – was evaluated in duplicate for BTZ-043 MICs to assess the extent of cross-resistance. Additionally, five BDQ-susceptible clinical strains carrying *Rv0678* mutations underwent duplicate MIC testing to determine the impact of these mutations on BTZ-043 susceptibility.

### Growth and fitness analysis

To assess the fitness costs associated with mutations in the *dprE1* and *Rv0678* genes, we monitored the growth of the *M. tuberculosis* H37Rv parental strain and seven selected *Rv0678* and *dprE1* mutants. This analysis was conducted in liquid 7H9 medium over a period of 3 weeks. The optical density at 600 nm (OD600) was measured at regular intervals to generate growth curves, using the methodology previously described in Ref. ^15^

### Ethical statement

Ethical approval was not required for this study as it involved the use of *M. tuberculosis* H37Rv laboratory strains and anonymised clinical isolates. All methods were performed in accordance with institutional guidelines.

## RESULTS

### Wild-type BTZ-043 MIC distribution

Based on 60 independent replicates of H37Rv, BTZ-043 MIC values were highly reproducible, with all replicates falling within a narrow three-dilution range (0.0005–0.002 μg/mL; Figure). In contrast, wild-type clinical MTBC isolates exhibited a broader MIC distribution, spanning five dilutions (0.00025–0.004 μg/mL), yet maintaining an overall mode of 0.002 μg/mL with no observable lineage effect (Figure).

**Figure.**
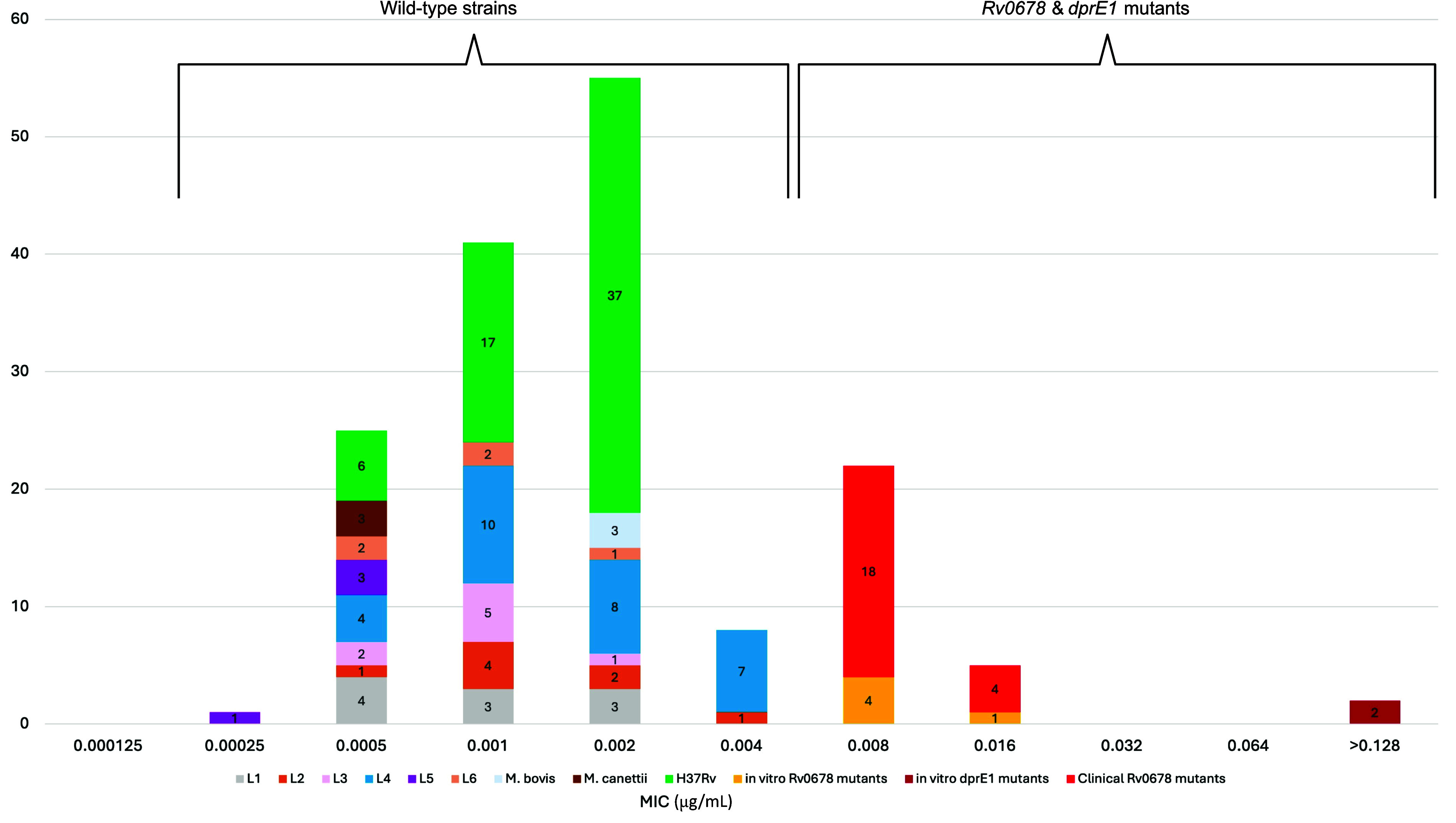
Distribution of BTZ-043 MICs in μg/mL for wild-type *Mycobacterium tuberculosis complex* strains compared with resistant mutants harbouring mutations in *Rv0678* and/or *dprE1*. Wild-type strains cluster at low MIC values, while mutants exhibit elevated MICs, indicating reduced susceptibility to BTZ-043. These data underscore the impact of *Rv0678* and *dprE1* mutations on BTZ-043 resistance by contrasting the MIC distributions of wild-type and mutant populations.

### Identification of resistance-associated variants

During the mutant selection process, we isolated more than 50 BTZ-043–resistant strains exhibiting potential on-target and off-target mutations. Subsequent WGS identified seven unique mutants. WGS analysis revealed mutations in two primary loci: *Rv0678* and *dprE1* (Table 1). *Rv0678* encodes a transcriptional regulator of the *mmpS5/L5* efflux pump, implicated in resistance to BDQ and CFZ.^10,11^ Mutations in *Rv0678* resulted in a 4- to 8-fold increase in the BTZ-043 MIC relative to MIC mode of H37Rv and wild-type clinical strains, suggesting a potential cross-resistance mechanism mediated by increased efflux pump expression. The mutation rate in *Rv0678* was approximately 1 × 10^−6^ to 4 × 10^−6^ on 7H10 agar containing 0.004 μg/mL BTZ-043. Conversely, Cys387Gly/Ser substitutions in *dprE1*, the primary target of BTZ-043, resulted in an MIC increase of over 1,000 fold on 7H10 agar containing the drug, underscoring the pivotal role of DprE1 in mediating BTZ-043’s anti-bacterial activity. Notably, no significant differences in growth dynamics were observed between wild-type and mutant strains during the exponential and stationary phases in the absence of BTZ-043 or other antibiotics (Supplementary Data Figure S1)

**Table 1.** Phenotypic and genotypic characterisation of the in vitro–selected BTZ-043-resistant mutants.

Strain	Gene			BTZ-043 MIC BMD (μg/mL)	BDQ MIC MGIT (μg/mL)	CFZ MIC MGIT (μg/mL)
	*Rv0678*	*dprE1*	*dprE2*			
M1	Met23Ile and Gln115_	WT	WT	0.008	4	2
M2	Ala62Val	WT	WT	0.008	4	2
M3	Ser68Gly	WT	WT	0.008	4	4
M4	Leu35Trp	WT	WT	0.016	4	2
M5	Gly25Ser	WT	WT	0.008	4	2
M6	Ala62Val	Cys387Gly	WT	>0.128	4	2
M7	Ala62Val	Cys387Ser	WT	>0.128	4	2
H37Rv ATCC 27294	WT	WT	WT	0.0005–0.002	0.5	0.5

### Impact of Rv0678 mutations on drug susceptibility

Sequential clinical isolates from six patients previously treated with BDQ-containing regimens exhibited a 4- to 16-fold increase in BTZ-043 MICs relative to baseline values, consistent with the emergence of *Rv0678* mutations (Table 2). BDQ-resistant clinical strains harbouring various *Rv0678* mutations exhibited a 4- to 8-fold increase in BTZ-043 MICs relative to the wild-type MIC mode, indicating that these mutations may contribute to reduced BTZ-043 susceptibility in the context of BDQ resistance (Table 3). However, MIC testing of BDQ-susceptible strains harbouring *Rv0678* mutations did not reveal a significant increase in BTZ-043 MICs relative to the wild-type MIC mode (Table 3).

**Table 2.** BTZ-043 MIC testing of sequential strains from patients developing an *Rv0678* mutation after receiving BDQ-containing regimens.^14^

	BDQ exposure (mo)	*Rv0678* mutation	Lineage	BDQ MIC (μg/mL) MGIT	CFZ MIC (μg/mL) MGIT	BTZ-043 MIC (μg/mL) BMD
Patient 1	0	WT	3	0.5	0.5	0.001
	9	48fs (779130_Ins-C)		4	4	0.008–0.016
Patient 2	0	WT	3	0.5	0.5	0.001
	5	48fs (779130_Ins-C)		4	4	0.008–0.016
Patient 3	0	WT	3.2.1	0.25	0.5	0.002
	6	Val20Gly		2	2	0.008
Patient 4	0	WT	1.1.2	0.5	0.5	0.001
	4	Glu104Lys		4	2	0.008
Patient 5	0	WT	3	0.5	0.5	0.001
	7	46fs (779125_Ins-G)		2	1	0.008
Patient 6	0	WT	4.9	0.5	1	0.002
	1	65fs (779181_Ins-G)		4	4	0.008

Interestingly, this has been seen also for BTZ-043, and all strains exhibited increased MICs compared with baseline, suggesting the emergence of low-level resistance or reduced susceptibility to BTZ-043.

**Table 3.** BTZ-043 MIC testing of BDQ-resistant and BDQ-susceptible clinical strains with different *Rv0678* mutations.

Strain ID	*Rv0678* mutation	BDQ DST	BDQ MIC (μg/ mL) MGIT	BTZ-043 MIC (μg/mL) BMD
344-20	46fs (779125_Ins-G)	Resistant	4	0.008
346-20	Arg156_ STOP	Resistant	2–4	0.008
347-20	Gly65Arg	Resistant	2–4	0.008
352-20	Ser52Phe	Resistant	4	0.008
353-20	Leu117Arg	Resistant	2	0.008
356-20	Gln76Arg	Resistant	2	0.008
360-20	Ala84Val	Resistant	2–4	0.016
362-20	44fs (779121_Ins_GT)	Resistant	2–4	0.008
363-20	6fs (779005_Del_G)	Resistant	4	0.016
380-20	Ala102Thr	Resistant	2–4	0.008–0.016
381-20	Leu142Arg	Resistant	2	0.016
382-20	64fs (779181_Ins_C)	Resistant	2	0.008
383-20	46fs (779125_Ins-G)	Resistant	4	0.016
386-20	Leu117Arg	Resistant	2	0.008
403-20	31fs (779082_Del_T) + Asn4Thr	Resistant	2	0.008
414-20	Arg50Trp	Resistant	2–4	0.008
402-20	Val120Met	Susceptible	0.5–1	0.002
378-20	Ser52Tyr	Susceptible	1	0.002
354-20	Arg90Cys	Susceptible	1	0.002
357-20	Val7Del (779005–779006_Del_GG)	Susceptible	1	0.002
404-20	Tyr157Ser	Susceptible	1	0.002

### Fitness cost of dprE1 mutations

Growth curve analysis of both wild-type and *dprE1* mutant strains in 7H9 medium showed no significant differences in growth during the exponential or stationary phases. The *dprE1* mutations did not result in any detectable fitness cost, as both the wild-type and mutant strains exhibited similar growth dynamics over the 3-week period (Supplementary Data Figure S1).

## DISCUSSION

This study elucidates new resistance mechanisms associated with BTZ-043, a promising anti-TB agent targeting the arabinogalactan biosynthetic enzyme DprE1. Our findings confirm that point-mutations in codon for Cys387 of the *dprE1* lead to a dramatic increase in BTZ-043 resistance, with MICs rising up to 1,000 fold. Mutations in *Rv0678*, a key regulator of *mmpS5/L5* efflux pump expression, were associated with reduced BTZ-043 susceptibility, resulting in a 4- to 8-fold increase in MICs relative to the wild-type MIC mode of 0.002 μg/mL. Our results corroborate findings from Poulton et al.,^7^ which indicate that *Rv0678* mutations can confer low-level resistance to BTZs. Similarly, Almeida et al.^8^ demonstrated that *Rv0678* mutations reduce susceptibility to another DprE1 inhibitor, A7371. These studies support our observation that while *Rv0678* mutations can impact BTZ-043 susceptibility, the extent of cross-resistance may vary. This variability underscores the complexity of resistance mechanisms and the need for ongoing surveillance to assess their clinical impact.

Elevated BTZ-043 MICs in sequential clinical strains from patients previously treated with BDQ-containing regimens^14^ indicate a potential for cross-resistance in clinical settings. This underscores the need for continuous resistance monitoring, particularly among patients with a history of BDQ or CFZ therapy. In our study, BDQ-resistant clinical strains harbouring various *Rv0678* mutations exhibited a 4- to 8-fold increase in BTZ-043 MICs relative to the wild-type MIC mode, suggesting these mutations may confer reduced susceptibility or low-level resistance to BTZ-043, especially in the context of existing BDQ resistance. Although *Rv0678* mutations are established contributors to resistance against BDQ and CFZ, their effect on BTZ-043 efficacy remains uncertain. Our data suggest that *Rv0678* mutations may lead to low-level cross-resistance to BTZ-043; however, the full clinical implications of this effect warrant further investigation. Interestingly, our study found that BDQ-susceptible strains harbouring *Rv0678* mutations did not exhibit increased BTZ-043 MICs (Table 3). This observation suggests that while *Rv0678* mutations can influence drug susceptibility, their effect is not uniform across all strains. Such variability may be attributed to differences in the specific mutations and their consequent impact on efflux pump expression levels.

The absence of a significant fitness cost associated with *dprE1* mutations indicates that these resistant strains may persist and propagate even in the absence of antibiotic pressure, potentially leading to widespread resistance. This observation aligns with previous studies demonstrating that *dprE1* mutations do not necessarily incur a substantial fitness penalty^10,16^ and underscores the need for vigilant monitoring during the clinical development and deployment of BTZ-043.

Given the potential for cross-resistance among BTZ-043, BDQ, and CFZ, it is imperative to optimise combination regimens to mitigate resistance. One promising strategy is to saturate the efflux pump by co-administering multiple agents – such as DprE1 inhibitors, BDQ, and CFZ – to overcome the resistance mechanisms conferred by *Rv0678* mutations. Such combination therapies may enhance treatment efficacy against MDR-TB strains while limiting the emergence of resistance. However, further studies are needed to evaluate the combined action of DprE1 inhibitors and BDQ, including investigations into their synergistic or antagonistic interactions in both in vitro and in vivo models, to determine the most effective treatment regimens and inform clinical practice.

Our study also underscores the need for diagnostic tools capable of detecting *dprE1* and *Rv0678* mutations in real time when BTZ-043 is incorporated into treatment regimens. Such diagnostics would guide therapeutic decisions by ensuring patients receive the most effective treatments while minimising resistance risk.^17^ Moreover, our findings highlight the importance of early assessments – such as establishing epidemiological cut-off values (ECOFFs), generating resistant mutants, and studying cross-resistance – during phase 2A/2B drug development. Early integration of these analyses supports the rational use of novel agents in targeted patient populations, aids in preventing resistance, and facilitates the development of tailored diagnostic tools. Future research should prioritise the optimisation of drug combinations to reduce selective pressure for resistance and improve treatment outcomes in TB patients.

This study’s limitations include the *in vitro* nature of the experiments, which may not fully capture the complexities of resistance development in clinical settings. Further research involving a broader range of clinical isolates from diverse geographical regions is necessary to better understand global resistance patterns associated with BTZ-043. Additionally, studies investigating the development of cross-resistance in both *in vitro* and *in vivo* models are essential to provide a more comprehensive understanding of resistance mechanisms.

## CONCLUSION

DprE1 inhibitors represent a promising class of novel anti-TB agents. However, the risk of resistance development, particularly in the context of concomitant BDQ and CFZ use, warrants careful consideration. Sustained research and rigorous surveillance are crucial to preserving the efficacy of DprE1 inhibitors, including BTZ-043, in the fight against TB.

## Supplementary Material


