# Supplementary material for: Low-level BTZ-043 resistance in Mycobacterium tuberculosis and cross-resistance to bedaquiline and clofazimine
Source: IJTLD Open. 2025 Oct 10;2(10):604–9. doi: 10.5588/ijtldopen.25.0301 (PMC12517268; doi:10.5588/ijtldopen.25.0301)
Supplement: Supplementary file 1 [file ijtldopen25-0301_supplementarydata1.pdf]

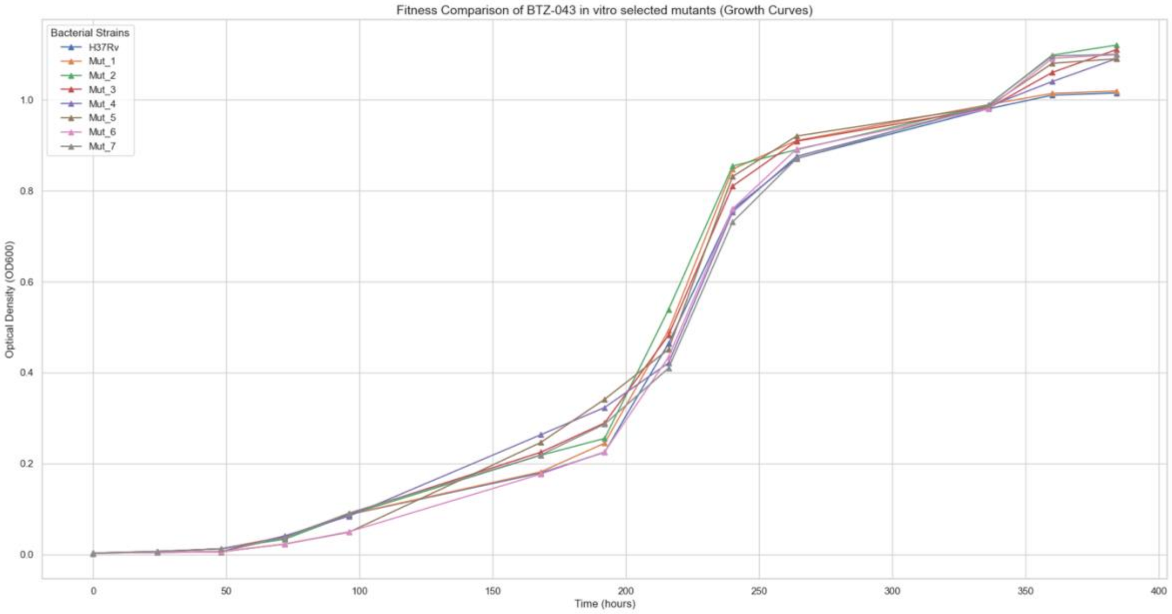

Figure S1: Growth Curve Analysis of H37Rv and *Rv0678/dprE1* Mutants in 7H9 Medium. This figure presents the growth curves of *Mycobacterium tuberculosis* strain H37Rv and seven mutants with modifications in the *Rv0678* and *dprE1* genes (Mut 1 to Mut 7) over a period of 350 hours. Growth was assessed in liquid 7H9 medium by measuring the optical density at 600 nm (OD600) at regular intervals. The depicted growth curves explore the fitness costs associated with these specific genetic alterations. The curves closely align, indicating similar fitness levels among the mutants under the experimental conditions.
